# Peer Review of “Beyond Expected Patterns in Insulin Needs of People With Type 1 Diabetes: Temporal Analysis of Automated Insulin Delivery Data”

**DOI:** 10.2196/66922

**Published:** 2024-11-27

**Authors:** Darlinton Carvalho

**Affiliations:** 1 Computer Science Department, Universidade Federal de São João del Rei, São João del Rei, Brazil

**Keywords:** multivariate time series, k-means, clustering, machine learning, temporal patterns, data-driven, OpenAPS, open dataset, type 1 diabetes, insulin needs

*This is a peer-review report for “Beyond Expected Patterns in Insulin Needs of People With Type 1 Diabetes: Temporal Analysis of Automated Insulin Delivery Data.”*

## Round 1 Review

### General Comments

This paper presents a study [1] about temporal patterns in insulin needs for type 1 diabetes. It employs various time series techniques to spot such patterns using matrix profiles and multivariate clustering. The OpenAPS Data Commons dataset, an extensive dataset collected in real-life conditions, was analyzed to discover temporal patterns in insulin needs driven by well-known factors such as carbohydrates and potentially novel factors. The results are limited to disclosing interesting temporal patterns in insulin need that cannot be explained solely by carbohydrates through the performed analysis. While patterns found are auspicious, they still lack scientific rigor and research into correlations and causalities that drive these patterns to truly inspire new research into type 1 diabetes.

### Specific Comments

#### Major Comments

1. The research is well-designed and developed.
2. Although the paper is well written and presents interesting results, it does not comply with the Instructions for Authors of JMIR [2].
3. The discussion and implications are minimal, leaving a more significant contribution to future work.
4. This article [3] is found in multiple publications on the internet.
